# Progress in Medicine

**Published:** 1897-11-06

**Authors:** 


### Progress in Medicine.

### DISEASES OF THE DIGESTIVE ORGANS.

**Gastric Ulcer.**—Professor Robin, of Paris,[1] in discussing the causes and diagnosis of hæmatemesis, enumerates first those forms of it in which bright blood is vomited. These are ulcer, cancer, cirrhosis of liver, hysteria, vicarious menstruation, varicose veins of the œsophagus, miliary aneurisms of the stomach, injuries and certain infectious diseases. Dark blood is brought up in cancer, in uræmia and strangulated hernia, heart affections, venous stases due to cancer elsewhere, yellow fever, purpura, and leucocythemia; but the distinction is not always an easy one. He rejects, as dangerous, Leube's treatment of ulcer by washing out the stomach and injecting bismuth in an emulsion; but when the first stages of the ordinary management under bismuth are past he advises the perchloride of iron as a valuable tonic and hæmostatic.

Ratgen[2] treats these ulcers by rectal nutrition alone without any drugs. For ten days the patient is kept in bed and fed solely by the rectum. Hot fomentations are applied if pain exists, and water is given for thirst. Alkalies are not given, because he thinks that when at rest the stomach secretes no acid. Like Robin, he does not allow a stomach tube to be passed.

C. O'Donovan,[3] while employing the ordinary methods, advises operative procedure when obstinate recurrent hæmorrhage occurs after apparent cure, especially in chlorotic and otherwise unhealthy individuals. Tuberculous ulcers of the stomach are very rare, but three cases are described by Dr. Alice Hamilton[4] in patients suffering from pulmonary disease. In two cases the ulcers were from 70 to 120 in number; in the third, two large ones only were present. Tubercle bacilli were found on the surface and imbedded in the tissues in every instance.

**Gastric Catarrh.**—T. M. G. Carter[5] finds that washing out the stomach with hot or cold douches containing bicarbonate of soda, or a 5 per cent. solution of hydrozone, is useful even in an early stage. Later on the wash may contain green soap as well. If washing out is impossible, he gives a draught of eight ounces of hydrozone (2 per cent.) before food, the patient lying first on his back and then on the right side, that the fluid may carry away the thick mucus from every part of the stomach. He advises a teaspoonful of glycozone after food, in addition to the ordinary treatment.

F. Murdoch[6] points out that we have to distinguish cases of this disease from cancer, ulcer, glandular atrophy, and neurotic dyspepsia. In the last-named affection the re-actions of the stomach contents may appear to be the same as in catarrh if a single examination only is made; but repeated tests showing the presence of enzymes and mucus and the absence of hydrochloric acid are found in catarrh alone. The absence of the rennet zymogen points to glandular atrophy. With regard to treatment, the writer gives little meat when the H.Cl. is absent, and no milk if rennet is no longer produced in the stomach. When salivary digestion fails taka diastase is useful. In other cases he employs only lavage, electricity, and the bitter tonics.

Dyspepsia nervosa is of favourable prognosis, and a test examination, says Rosenbach,[7] may prove to the patient that he has no organic disease. Hot water and fomentations may be given for pain, and small doses of opium or belladonna on an empty stomach will be useful, and when there is great hyper-æsthesia even cocaine may be desirable.

Mental treatment is, of course, important, and eating soon after any mental excitement should be forbidden. In these nervous cases taka diastase has no effect, but Friedenwald[8] remarks that in catarrh with subacidity it increases the acid and promotes the digestion of starches. In hyper-acidity it actually decreases the acid and increases the motor power and the starch digestion, while in motor insufficiency it remedies that defect without any other action. These results, founded on exact experiments, are of great importance, and show the wide field for the use of this substance. Leo[9] confirms his view of its value in hyper-acidity, when alone or when complicated with ulcer. The pain and constipation are often remarkably diminished. To obtain the best results all starch foods should be taken at the beginning of the meal.

A fermentation of proteids with the evolution of $H_2S$ has been referred by Strauss[10] to the action of bacillus coli, and the curious fact was observed that the addition of sugar or other carbohydrates to the diet led to the production of lactic acid instead of $H_2S$, the microbe probably finding a more easily assimilable food and leaving the proteids untouched.

**Reichman's Disease.**—Some writers recognise two forms of this chronic gastritis with hyper-acidity—the continuous and paroxysmal ones. The former is referred by Hayen to stenosis of the pylorus, while Doyen[11] holds that the hyper-secretion is primary, and is followed by pyloric spasm. Mathieu notices[12] the following types of hyper-secretion: (*a*) That which occurs in perfect health in a few individuals without any symptoms whatever; (*b*) neurotic gastric crises accompanied by hyper-secretion and dependent on some nerve disease only; (*c*) similar attacks due to fatigue or poisonous food without any continuous hyper-acidity; (*d*) permanent hyper-secretion with much pain three or four hours after food; (*e*) this may be accompanied with stasis or preceded by an organic stenosis; (*f*) lastly, it may be complicated with the presence of a round ulcer. It is clear that the treatment must depend on the cause of the condition in the special case. Where we have simple hyper-secretion with pain, he gives alkalies, preferably magnesia or chalk, in quantities sufficient to stop the post-prandial pain, and at first a milk diet. Le Gendre insists on the importance of mental rest and warm baths in the neurotic cases, while Soupoult believes that in many instances there is a primary hyper-æsthesia which may cause all the symptoms without necessarily going on to an excessive secretion of acid.

**Dilatation.**—Einhorn[13] reports ten cases of benign stenosis of the pylorus operated on surgically of which eight were cured, while great relief was obtained in malignant cases by the same procedure. Thomas finds[14] that not infrequently pyloric obstruction is due to adhesions following gall-stones, ulcers, and other causes; and as symptomatic of this condition he notices that when the stomach is insufflated the pylorus will remain fixed at the edge of the ribs if an adhesion exists. Again, if the patient walks about, vomiting is much more frequent than when he is kept in bed, and is brought on at once if a full meal is taken. Surgical treatment may sometimes be necessary, and may take the form of gastenterostomy or of breaking down adhesions.

**Cancer.**—Hammerschlag,[15] in discussing the early diagnosis of this affection, concludes that even if a tumour and wasting are absent we can still come to a positive decision if in several examinations spread over three or four weeks we find hydrochloric acid always absent, the proteid digestion reduced below 15 per cent., with much lactic acid and many long bacilli. Hæmatemesis and loss of motor power are also of importance, but no single sign in itself is reliable. Thus, Benedict has observed the absence of hydrochloric acid in fifty cases where no cancer existed.[16] Lactic acid is of still more uncertain significance, and the occasional deficiency of urea excretion is not more marked than in several other diseases. A very interesting case of cancer in a male of 20 years is given by George Bock, and it appears that several other observers have found it during early life, contrary to the usual belief. It is then, however, generally of very slow growth, and not easy to diagnose, because cancer of the intestine is so frequent at this age.

In this paper are given a large number of drawings of cells which have undergone indirect nuclear division, and contain many nuclei and fatty granules. These are regarded by Eickhorst as diagnostic of cancer, and Rieder has been able by their means to diagnose the presence of a growth which he verified post mortem. Still, it seems very doubtful whether similar atypical mitoses do not also occur in other pathological states. Certainly, we can not rely upon any one typical cancer cell, as the older observers thought to be possible.

[1] Med. Week, April 7. [2] Amer. Jour. Med. Sc, April. [3] New York Med. Jour., July 10. [4] Johns Hopkins Hosp. Rep, April. [5] Amer. M.S. Bulletin, May 10. [6] New York Med. Jour., Aug. 28. [7] B. Med. Jour., July 20. [8] New York Med. Jour., May 29. [9] Amer. Jour. Med. Sciences, April. [10] Amer. Jour. Med. Sciences, June. [11] Practit., August. [12] Amer. M.S. Bulletin, July 10. [13] Med. Rec., July 10. [14] B. Med. Jour., June 5. [15] Amer. Jour. Med. Sciences, April. [16] Inter. Med. Magaz., April.

### OBSTETRICS.

**Ectopic Gestation** (*Continued from page* 82.)—Freeland Barbour[9] read notes of a case where considerable difficulty in diagnosis had arisen between ectopic gestation and retroversion of the gravid uterus; the patient was operated on eleven months after the last discharge of blood, which was probably associated with the primary rupture, but was thought to be an ordinary period. Another case was operated on nine months after the last normal menstruation, and a mummified five and a half months' fœtus extracted; the cyst wall was stitched to the abdominal wall.

Arthur Giles and Ewen Maclean[10] described two cases which presented the symptoms and physical signs of retroversion of the gravid uterus, one with retention of urine. The first case was complicated by chronic intestinal obstruction. In both cases attempts were made to push up the mass under an anæsthetic. Two conclusions were drawn as to treatment: first, that a pelvic tumour, differing in signs and symptoms from ordinary types, should be dealt with surgically and not by the expectant method; and secondly, that early operation was always advisable. In support of the second proposition, Coplin Stinson[11] insists that early operation may prevent rupture, and that therefore operation should take place as soon as the diagnosis can be made, but if rupture has occurred there should be no delay. He emphasises the fact that the short way of staying hæmorrhagic collapse is to arrest the hæmorrhage, and strongly recommends this course instead of waiting for reaction, which latter has undoubtedly cost several women their lives.

Grandin[12] also insists on the advantage of immediate operation and rapid checking of hæmorrhage. A case of ruptured interstitial pregnancy treated by suture of the fundus is reported by Tytler.[13] Rupture of the tube in its interstitial portion is rare, and the treatment advised in the absence of the possibility of securing the pedicle has been by complete hysterectomy. At the operation the peritoneum was found to be full of black clotted blood; there was no sign of recent bleeding, and none occurred on clearing out the clots. A rent was found in the fundus of the uterus, extending from the middle of the fundus to the origin of the right Fallopian tube; the length was nearly 2 inches, the right side of the uterus being much enlarged. After the removal of the right tube and ovary the rent was closed with fishing-gut sutures. The patient made a good recovery.

A somewhat similar case was reported by Kynoch.[14] In this case the operation was not performed for symptoms of rupture, but for abdominal pain, and other distressing symptoms. Part of the cyst in which the fœtus lay was formed by the uterine wall, in which was a cavity. No pedicle could be ligatured in such a way as to check hæmorrhage, so the upper part of the uterus was removed, and a stump formed by suturing peritoneal and mucous surfaces respectively together. Secondary hæmorrhage occurred, and the wound was reopened, and the rest of the uterus removed. The patient, however, died.

The treatment of ectopic pregnancy by vaginal puncture is dealt with by Kelly.[15] He first treated in this way a case occuring in a patient too weak for laparotomy. The sac was punctured by scissors through the lateral fornix, the clots turned out, and a drainage tube inserted. The patient recovered. In another case, finding, on performing laparotomy, that dense adhesions prevented the removal of the sac, he made a free opening into it through the vaginal vault, and drained it. Recovery followed

He operates by plunging upwards a pair of sharp scissors at a point behind the cervix in the vaginal fornix close to the sac. The scissors are opened, and withdrawn open. A larger pair, if necessary, are then introduced and withdrawn in the same way until two fingers can be introduced. The sac is then emptied completely down to its shell, and surrounding adhesions. The sac is then irrigated and plugged with gauze, which is removed after some days, and the sac washed out daily until it closes. The chief danger is from hæmorrhage from the degenerated villi, which occurred to such an extent as to necessitate laparotomy in one case; in another, a douche nozzle was thrust through the sac, and about two quarts of boric solution injected into the peritoneal cavity; this also necessitated laparotomy, but did not prevent an excellent recovery. He treats pelvic suppuration by the same method.

**Ante Partum Hæmorrhage.**—Hæmorrhage occurring before labour or at its commencement demands alike a correct knowledge of the pathological reasons for its occurrence and the practical means to be applied to check it.

Hofmeier,[16] in discussing placenta prævia, states that hæmorrhage comes on earlier in cases of partial or marginal implantation than in cases in which the implantation is central. He attributes this to the greater ease with which the free edge can slide upon the uterine wall when a part of it is already over the cervix, and so partially detached, than when the edges are firmly attached all round as in the central variety. He does not lay stress on the anatomical difference between true placenta prævia and hæmorrhage caused by low implantation, since the treatment of the hæmorrhage is the essential point in each case. He recommends plugging the vagina firmly with aseptic gauze, following this by the use of a colpeurynteur introduced into the cervix, followed up, after dilatation is sufficient, by rupture of the membranes, version, and the drawing down of a leg. He has used this method in every case of placenta prævia, and found it more successful than any other. Füth[17] disapproves of the tampon and of expectant treatment in any form; he also uses the colpeurynteur, but makes traction on it. His statistics (50 cases) show a mortality of 38 per cent.

**Delivery in Contracted Pelvis.**—The treatment by Pinard of pelvic deformity obstructing delivery is described by Sinclair.[18] The cases occurred at the Baudelocque Clinique, and numbered 95. In 27 of these delivery was effected artificially; forceps were used 6 times, version once, craniotomy 4 times, each time after the death of the child. Symphysiotomy was performed 14 times; in 13 cases the pelvis was rachitic; in 1 rachitic, with old hip disease. Extraction was completed by forceps in 13 cases; version in 1. Two mothers died. All the children were born alive, but 4 died within 15 days of birth; 1 death occurred from acute pneumonia and 1 from sepsis, but in neither case was the fatal issue attributed directly to the operation. In every case the symphysis united readily, and the patients resumed their occupations.

Pinard has in 12 cases performed the operation a second time. Symphysiotomy should not be performed until the soft parts are fully dilated. During the past year 5 patients who have previously undergone symphysiotomy have been delivered of living children spontaneously, which appears to show that slight permanent increase of the diametus remains after the operation.

The statistics of symphysiotomy given above should be contrasted with those of induced premature labour given by Warden[19] from 39 cases in the Glasgow Maternity: Maternal deaths—Induced labour, 1, or 2·56 per cent.; symphysiotomy, 14·2 per cent. Children born alive—Induced labour, 24, or 66 per cent.; symphysiotomy, all, 100 per cent. Children died within 15 days—Induced labour, 10, or 25·6 per cent.; symphysiotomy, 28·5 per cent.

Another method of facilitating delivery in cases of contracted pelvis is by placing the patient in the Walcher position, *i.e.*, the breech at the edge of the bed or table, with the legs fully extended and hanging. This causes a certain amount of increase of the conjugate diameter by causing rotation of the ossa innominata on the ilio sacral articulation. Jardine[20] describes a case in which he extracted, in this position, with axis traction forceps, a fœtus weighing 8½ lb.; the conjugata vera, as estimated by Jardine and two other observers, would not be more than 2¾ inches.

**Breech Presentation.**—In a paper on the dangers and diagnosis of breech presentations Spencer[21] states that from 20 per cent. to 50 per cent. of children presenting in this way are stillborn. To avoid this large mortality he recommends the routine use of abdominal palpation, to diagnose the position of the child at about the seventh month of pregnancy if the breech is found to be presenting, the substitution of the head by external version, and the maintenance of the cephalic presentation by the wearing of a belt.

[9] B. M. J., Jan. 23, 1897. [10] B. M. J., July 17, 1897. [11] Therapeutic Gazette, April 10, 1897. [12] Med. Record, loc. cit. [13] B. M. J., June 12, 1897. [14] B. M. J., May 22, 1897. [15] Lancet, April 3, 1897. [16] Medical Week, June 25, 1897. [17] Centralblatt für Gynak, quoted in N. Y. Med. Record, May 15, 1897. [18] Med. Chron., April, 1897. [19] Edinburgh Med. Jour., Jan, 1897. [20] Glasgow Med. Journ., April, 1897. [21] Lancet, Feb. 27, 1897.

